# Therapeutic use of music in neurological disorders: A concise narrative review

**DOI:** 10.1016/j.heliyon.2024.e35564

**Published:** 2024-08-08

**Authors:** Medha Ramaswamy, Johann Laji Philip, Vijayan Priya, Snigdha Priyadarshini, Meenakshi Ramasamy, G.C. Jeevitha, Darin Mansor Mathkor, Shafiul Haque, Fatemeh Dabaghzadeh, Pratik Bhattacharya, Faraz Ahmad

**Affiliations:** a Department of Biotechnology, School of Bio Sciences and Technology (SBST), Vellore Institute of Technology, Vellore, 632014, India; b Department of Biosciences, School of Bio Sciences and Technology (SBST), Vellore Institute of Technology, Vellore, 632014, India; c Research and Scientific Studies Unit, College of Nursing and Allied Health Sciences, Jazan University, Jazan, 45142, Saudi Arabia; d Gilbert and Rose-Marie Chagoury School of Medicine, Lebanese American University, Beirut, 1102 2801, Lebanon; e Centre of Medical and Bio-Allied Health Sciences Research, Ajman University, Ajman, United Arab Emirates; f Department of Clinical Pharmacy, Faculty of Pharmacy, Kerman University of Medical Sciences, Kerman, Iran; g School of Architecture, Vellore Institute of Technology, Vellore, 632014, India

**Keywords:** Schizophrenia, ADHD, Parkinson's disease, Dementia, Cognition, Circadian rhythm

## Abstract

**Background:**

Music elicits multifactorial benefits in emotional, social, cognitive, and academic aspects of human life. Music is clinically proven to reduce stress and anxiety, and improve mood and self-expression, particularly after traumatic events. Studies have also demonstrated that music promotes parasympathetic autonomic systems, suppresses hyperactivation of stress responses, and boosts immune functions. However, its ability to promote brain plasticity and signalling are only beginning to be realized. Moreover, its employment as a therapy for the treatment of specific aspects of other neurological disorders, including neurodevelopmental and neurodegenerative conditions and their comorbidities, is fast becoming an interesting field of research.

**Objective:**

The aim of this review is to summarize some of the recent studies focused on evaluating the applications of music therapy. For this purpose, we have focused on disorders encompassing both temporal extremities of brain developmental stages, from developmental conditions of autism and attention deficit hyperactivity disorder (ADHD), to ageing-related pathologies of Parkinson's disease and dementias.

**Results:**

The findings of the reviewed studies indicate potent utilities of music-based interventions in beneficially affecting multiple spheres of brain functions, such as sensorimotor, auditory, communication/language, psychological/emotional, behavioural, sleep and memory and cognitive attributes of patients diagnosed with diverse neuropathologies. Nevertheless, lack of standardized protocols for music provision as well as absence of information regarding key aspects, such as cultural and musical orientations of subjects and therapists'/caregivers' attitudes, have hindered the complete realization of music's therapeutic potential for neurological conditions. Further, while some studies have undertaken assessments of core neurophysiological mechanisms underlying music therapy, this information is largely lacking for most clinical studies.

**Conclusion:**

While this is not an exhaustive review of literature, we do hope that it serves as a platform to promote future research for establishing music therapy as a relevant neurotherapeutic strategy.

## 1 Introduction

In recent years, with the prevalence of detrimental life style changes and increased life expectancies, coupled with enhancements in medical technologies, incidences of some of the most prevalent neurological conditions have been coming to forefront. These include dysfunctions during the entire developmental spectrum, from neurodevelopmental pathologies, such as schizophrenia, autism and attention deficit hyperactivity disorder (ADHD), to age-induced neuronal deficits encompassing Parkinson's disease, dementia and neuropsychological dysfunctions. Multiple research groups and organizations have directed their efforts in diagnosis and therapy of these disorders. Music therapy has emerged as an interestingly non-pharmacological lifestyle intervention with potential multimodal benefits for these neuropathologies (Table 1). The primary aim of this review is to critically analyse the relevances of music therapy in the treatment of these specific disorders and their comorbidities, citing suitable examples from recently conducted research studies. We first begin our discussion with the influences of music as a therapeutic intervention in stimulating brain plasticity and signalling. Next we attempt to clarify the beneficial roles of music therapy in traumatic brain injuries (TBI). Lastly, we assess the abilities of music therapy in providing specific, but multimodal enhancement of brain functions in neurodevelopmental and ageing-related pathologies in a disease/condition specific manner.

**Table 1:** Music-based interventions in different nuerological disorders and their pathophysiological effects.

INTERVENTION	NEUROLOGICAL DISORDER/ASSESSMENT GROUP	OBSERVED EFFECT	REFERENCE
Rhythmical “functionally-oriented music therapy” and “music-supported training”, including assisted music playing using instruments such as drums and piano	Traumatic brain injury	Improvements in executive functions and increases in the grey matter volumes of the right inferior frontal gyri, improvements in specific cognitive processes and fine motor skills, beneficial neuroplasticity changes in the recovering brain of TBI patients	[75]
Long-term vocal musical expression (voice- and piano-based)	Right handed vocalists and pianists	Strengthening of white matter networks related to emotional regulation, voice control, sensory perception and language	[8]
Rhythmic auditory stimulation (RAS)	Traumatic brain injury	Enhanced performance in Functional Gait Assessment (FGA), indicating beneficial alterations in the spatio-temporal aspects of gait and reduced risk of falls	[16]
Listening to different kinds of music (classical, pop, rock, and heavy metal genres) via wireless headphone	Parkinson's disease	Genre-specific effects of music on spatio-temporal parameters of gait and trunk kinematics (e.g., music belonging to classical genre diminished walking speed and trunk tilting, while rock and heavy metal enhanced pelvic movement)	[18]
Group instrumental music therapy	Dementia-related cognitive impairment	Enhancement of social and behavioral attributes, such as peer and staff interaction, communication of emotions, daily-life activities, motivation, etc.	[27]
Musical tracks in English (rock, metal, electronic and rap) and Urdu (patriotic, melodious, qawali and ghazal)	Psychological stress and anxiety	English music tracks likely have comparatively more effectiveness in decreasing stress level, and females show enhanced sensitivity of music to reduce stress	[36]
Child-centric musical intervention involving instruments, songs and rhythmic cues	Children (6–12 years) with autism spectrum disorder	Significant positive impacts on functional audition-related brain connectivity, as well as parent-reported social communication	[37]
Calm music (with and without lyrics), and rhythmic music with lyrics	Attention-deficit hyperactivity disorder	Improvements in attentive (reading, comprehension) abilities in ADHD, but not in typically-developed pre-adolescents	[41]
Mozart's piano sonata, K.488 and D major (12 h/per day from post natal day 21–76)	Sprague-Dawley rat pups exposed to maternal separation-induced early life psychological stress	Reversal of social and psychological dysfunction induced by early life stress, resulting in significant enhancements of social interactions, and reductions in anxiety- and depression-like behavior, in addition to enhanced mature dendritic spine numbers in hippocampal CA1 neurons	[46]
Mozart's piano sonata, K.488 at 65–75 dB (8 p.m.–10 p.m./day for 3 weeks)	Juvenile Sprague-Dawley rats tested for anxiety-like behavior post music ecposure	Attenuation of anxiety-like behavior, facilitation of fear extinction, and enhancement of BDNF levels	[47]
Classical Indian music (stable/no variations, and with incremental variations in tempo and octave)	Undergraduate medical students	“Varying music” specifically decreased anxiety scores, and stimulated switching of heightened to reduced mind wandering states	[48]
Mozart's sonata K. 448 via a stereo system	Schizophrenia	Increased functional connectivity between pallidum and ventral hippocampus, and the striatum-default mode network circuitry, reduction in the negative schizophrenic symptoms	[57]
Emotional instrumental musical (sad/happy) stimuli (excerpts of 12 s durations)	Adults with autism spectrum disorder	Increased neural activity in dorsolateral prefrontal brain regions in response to happy vs. sad music in ASD subjects, possibly indicating enhanced cognitive processing and physiological arousal	[70]
Receptive (listening) and active music, 50 min sessions, twice a week, for 3 months (a total of 24 sessions)	Children and adolescents with attention-deficit hyperactivity disorder	Increased levels of serotonin, reduced levels of cortisol, in addition to improved psychological attributes of depression (as assessed by Children's Depression Inventory; CDI) and stress (as assessed by Daily Hassles Questionnaire; DHQ)	[72]
Group music therapy sessions (50 min, twice weekly, for 5 weeks)	Children (6–9 years) with autism spectrum disorder	Enhanced social skills (assessed by Social Responsiveness Scale; SRS), improved joint attention and eye gaze towards peers and other people	[76]

According to the American Music Therapy Association (AMTA), music therapy is a validated health-related discipline that utilizes music as an ameliorative regimen for addressing cognitive, social and psychological requirements. It involves employment of music for the achievement of personalized goals, including attenuation of stress-, anxiety- and depression-like behaviours, and upliftment of mood and self-expression. Indeed, the scope of music therapy is very wide, encompassing cognitive, academic, psychological, social and communicative qualities of human life [1]. Additionally, music serves as a simple distraction from distressing events, such as medical procedures. Moreover, music as a non-pharmacological agent has a long history of being used as a coping mechanism for distress and aiding in the healing process after exposure to painful and/or traumatic events [2].

**Fig. 1:**
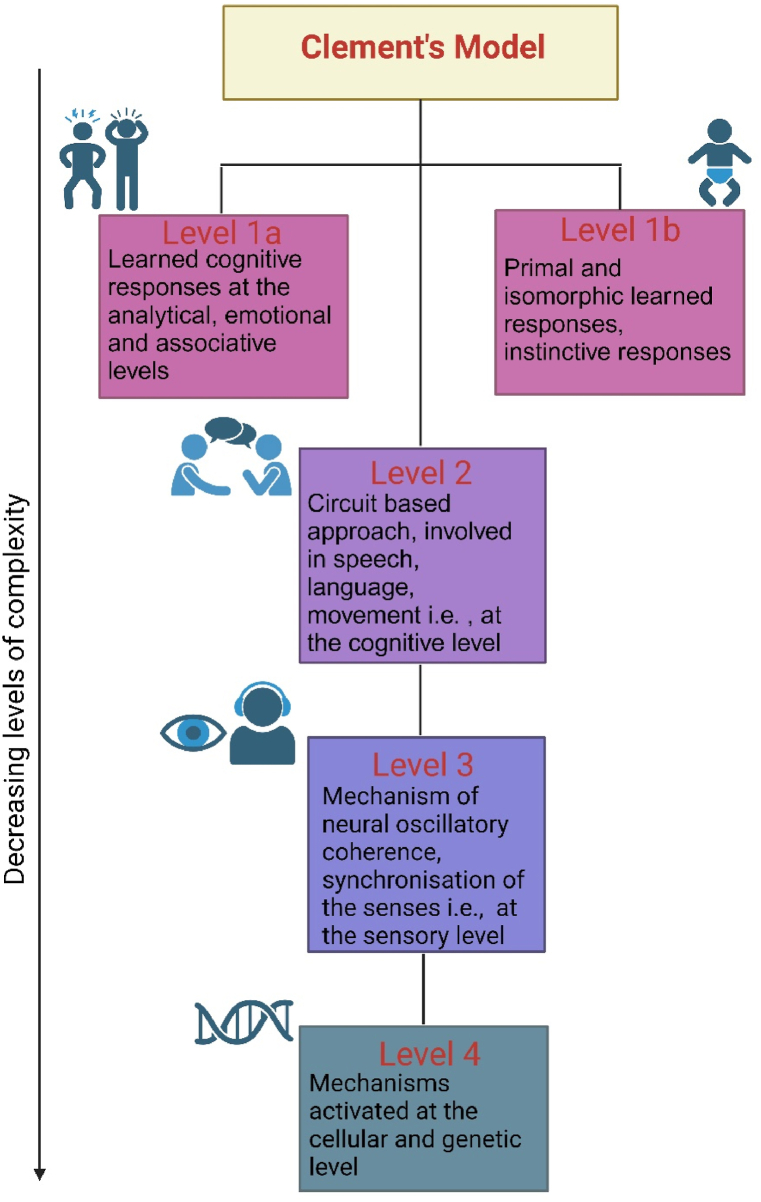
Clement's model of responses to musical stimuli. Clement's model is depicted as a four step scheme, arranged in decreasing levels of complexity. The first level is learned cognitive response and primal and isomorphic learned responses. The second level is circuit based approach. The third and fourth levels comprise of mechanisms at sensory, and cellular and genetic levels, respectively.

Music therapy is a multidisciplinary field and requires trained therapists for administering it. Although the positive psychological effects of music therapy are well-founded, the precise underlying mechanisms remain largely obscure. In this regard, studies on rodents indicate that specific musical stimuli lead to a shift toward stimulated parasympathetic autonomic activity, diminished endocrine stress responses, and enhancement of immune functions [3]. A quadripartite model of responses to musical stimuli has been proposed by Bernatzky et al., during their exploration of music therapy as pain mitigating strategy [4]. As depicted in Fig. 1, level 1a involves triggering poignant emotions linked to specific memories, prompting event-associated actions. Level 1b deals with primordial responses to music, rooted in early heart rhythms and associated emotions. Cognitive activation of neural circuits is dealt under level 2, while level 3 and 4 delves into stimulation of neural coherence and cellular and genetic aspects, respectively.

The study received an exemption from an Institutional Review Board/Ethics committee since no primary data were generated, and it did not involve human participants.

## 2 Music and brain plasticity

Brain plasticity, also known as neuroplasticity, is the ability of neurons and other cells of the nervous system to alter its morpho-functional characteristics in response to internal and/or external stimuli. Likewise, synaptic plasticity pertains to the capacity of the nervous system to modify synapses, the major pathways of interneuronal communication. Synaptic plasticity plays crucial roles in determining almost all aspects of higher-order brain functions, including learning and memory, execution and planning, and social and emotional behaviours.

**Fig. 2:**
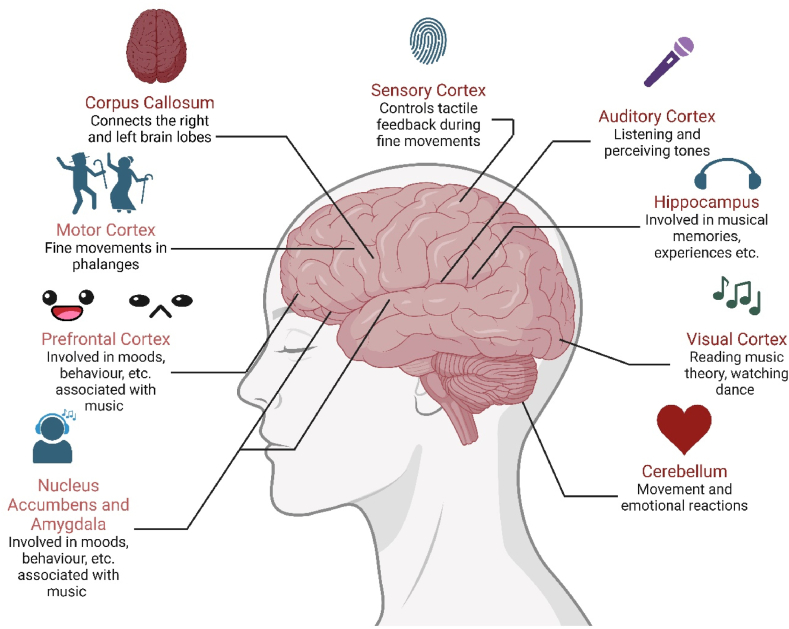
Summarization of the brain region-specific effects of exposure to music. The diagram depicts region-specificity of therapeutic aspects of music as an animation of the human brain. The different parts of cortices contribute to therapeutic effects at different levels; viz. Somatosensory, visual, auditory, motor and mood. Hippocampus is involved in memory-related aspects, while cerebellum contributes to motor responses. Nucleus accumbens and amygdala participate in emotional and behavioural aspects of music therapy.

The relationship between music and brain plasticity has been extensively studied due to the multidimensional aspects of visual, motor, and auditory cues. Favourable changes in the physiology of neurotransmitters (e.g., serotonin, dopamine, oxytocin, glutamate, etc.) involved in social and emotional aspects of behaviour, and cognitive functions of attention, imagination and creativity can be induced as a response to sensory perception of music [5]. Music also influences brain plasticity at multiple brain region levels (Fig. 2), stimulating the neuronal connections between the association cortices, and aiding in the cognitive processes of multisensory perception and responses against them [6]. Such music therapy-associated brain plasticity is also observed in volume increases in the grey matter of multiple sub-regions of cerebral cortices of clinical cases of traumatic brain injury (TBI, section 3) upon exposure to music-based therapeutic interventions, which have been proposed to be responsible for the significant improvements in the executive, attention, and imaginative aspects of cognition in these subjects [7]. Lastly, white matter neuroplasticity has been proposed as another substrate of music-based interventions. Such plastic changes in critical brain regions such as amygdala and motor cortices, may underlie music's beneficial effects on sensory processing and feedback, communication and emotional health in an experience-dependent manner [8]. Refinement of white matter neuroplasticity in right dorsal, corpus callosum, thalamic and corticostriatal pathways may underlie music's beneficial effects on executive functions in TBI subjects [9].

**Fig. 3:**
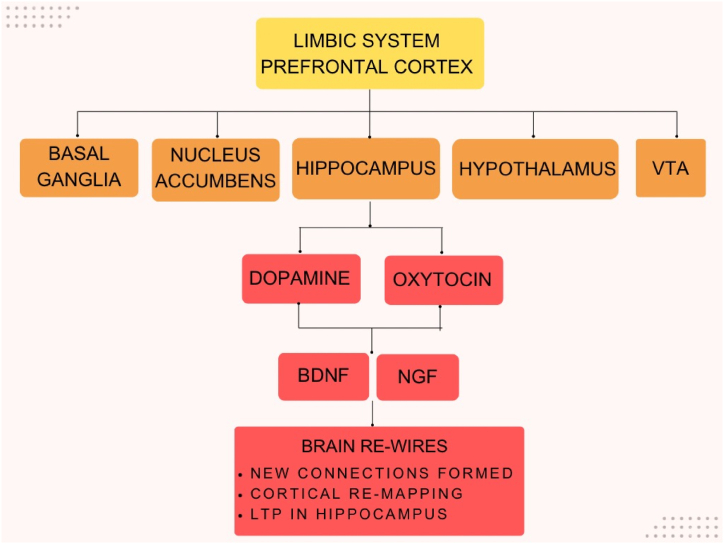
Proposed neurobiological mechanisms underlying music therapy. The figure shows a flowchart of mechanisms underlying neurotherapeutic aspects of music. Limbic system and prefrontal cortices relay the information to sub-regions of basal ganglia, nucleus accumbens, hippocampus, hypothalamus and VTA. This information is converted to molecular responses in form of enhanced dopamine and oxytocin signalling, resulted in stimulation of neurotrophic factors (BNDF and NGF), which finally cause enhancement in neuronal connections and their strengths.

Music perception is associated with numerous beneficial effects on sensorimotor, cognitive and emotional processing. Individuals may associate “good” music with “musical chills”, which are characterized by stimulation of the responses of the autonomic nervous system, often associated with enhanced heart rate, and decreased temperature and blood volume pulse [10]. Such musical chills are associated with dopaminergic reward response regions of the hypothalamus and amygdala, activation of which signifies intense emotions and pleasure [11]. Some of the molecular and cellular players implicated in music therapy's positive impacts on brain plasticity are outlined in Fig. 3. Additionally, readers are directed to a recent review by Chatterjee and colleagues [12], which comprehensively details the multimodal effects of music interventions on brain plasticity and rewiring with regards to diverse neuronal systems, including memory and cognition, emotional, behavioural and reward pathways, and sensorimotor, auditory, and language networks.

## 3 Traumatic brain injury (TBI)

TBI occurs when there is damage to the brain due to a sudden, mechanical assault. Pathophysiology of TBI is characterized by primary and secondary phases of vascular, axonal and neuronal degeneration, and may be associated with adverse effects on the individual's physical, mental, emotional, and cognitive health. As one of the more recent therapies for TBI, music has shown to have promising effects. Music therapy in TBI is linked to evocation of brain activity and reformation and/or restoration of damaged resting state networks [13]. Cognitive attributes, such as executive functions of TBI subjects have also been elicited to be significantly improved via music-based interventions. In particular, rhythmic music is associated with increase in neuroplasticity and grey matter volumes [13]. Beneficial effects of neuronal plasticity mechanisms have been proposed as pathways for music therapy-based stimulation of sensorimotor, emotional and cognitive functioning of TBI subjects [14]. Particularly, deficits in attention and memory domains of cognition have been reported to be robustly attenuated by neurologic music therapy regimens [15]. Music therapy has also been linked to significant improvements in motor deficits following TBIs and spinal cord injuries (SCIs). Thompson and co-workers reported that rhythmic music-induced improvements in multiple spatio-temporal attributes of gait, including speed, symmetry and cadence [16]. A systematic analysis of primary research indicated that music-based interventions had positive impacts on gait and gait speed in clinical subjects of CNS injuries [17]. While rhythmic music may be associated with increased activation and stimulation of auditory-motor networks, more studies are warrantied to reaffirm these aspects of music therapy.

## 4 Ageing-related neuropathologies: Parkinson's disease and dementia

Parkinson's disease is a degenerative condition characterized by progressive loss of dopaminergic neurons in the motor-associated brain regions, culminating into severe movement dysfunctions, particularly those related to repetitive automatic muscle functions [18]. Musical stimuli have been proposed as therapeutic interventions in alleviating Parkinson's disease-associated symptoms of motor imbalance, discoordination, rigidity, and slowness [19]. Indeed, a recent systematic review of literature affirmed the effectiveness of music therapy in inducing improvements of motor functions, cognition, communication, mental health status and spatio-temporal reasoning in clinical subjects diagnosed with Parkinson's disease [20].

With regard to motor functions, singing has been observed to stimulate gait and overall motor performance in both Parkinson's disease cases and aged adults [20]. However, it should be noted that rhythmic music, in complementation with dancing, is heavily dependent on the severity of the disease and individual differences [21]. Interestingly, Boni and Cattaneo have reported that exposure to music-based therapeutic regimen developed by Helvetic Music Institute in Parkinson's disease and dementia patients resulted in significant improvements in fine and gross motor skills, emotional and memory and cognitive attributes, as well as enhanced quality of life [22]. Communication deficits are another key aspect of Parkinson's disease. In this regard, intervention-based music has beneficial effects on speech-related neural networks and pathways. Indeed, ‘ParkinSong’ as a musical intervention has been elicited to improve speech and communication in human subjects with Parkinson's disease [23]. Interestingly, group singing and writing sessions for PD subjects and their spouses has been proposed to as a tremendous strategy for emotional upliftment and stimulation of couple relationships [24]. Along similar lines, Hayes et al. [25] integrated music therapy sessions based upon melodic intonation therapy, rhythmic auditory stimulation, singing and vocal exercises, and song writing, etc. In treatment regimens for aged subjects suffering from stroke, Parkinson's disease, or dementia. Their findings indicated that music therapy resulted in significant improvements in motor functions of the patients, in addition to beneficial effects on speech and communication [25]. Mood and emotional upliftment and improvement of mental health variables are other key attribute of music therapy in Parkinson's disease [20]. Lastly, in addition to the direct impacts on motor and communicative skills, music therapy has been evidenced to serve as a cognition- and memory-enhancing strategy for Parkinson's disease subjects [26]. In conclusion, music, in combination with dance therapy appears to be a robust non-pharmacological intervention for alleviating multiple dysfunctions associated with Parkinson's disease patients, helping them regain joy from functional activity, improve body and soul integrity, and restore positive self-acceptance and autonomy.

Dementias represent progressive decline in a plethora of cognitive abilities including memory, planning, executive, and social skills. In addition to amnesia, demented individuals often elicit symptoms of confusion, mood swings, difficulties in communication, changes in personality, and hampered daily activities. Several factors have been linked to the development and progression of dementias in humans. Considering the varied pathological mechanisms, dementias can be categorized into multiple types. Some of the most prevalent dementia types include Alzheimer's disease, vascular dementia, Lewy body dementia and fronto-temporal dementia. In the absence of effective disease preventive regimens, symptomatic treatments often involve lifestyle changes, in combination with other forms of therapies, such as music-based interventions.

Indeed, music-based therapy is one of the lifestyle interventions which has been found to be particularly effective in improving patient outcomes of dementia subjects [27]. A study conducted by Moreno-Morales and co-workers confirmed music therapy's positive impacts on cognitive status, expressive states, and self-awareness, with consequential enhancement of quality of life in dementia subjects. Activation of brain areas associated with cognition, in addition to circuitry for perceiving sounds, rhythms, lyrics, and patterns were observed in these subjects [28]. Mood enhancement and attenuation of behavioural problems have also been reported to be significant facets of music therapy in dementia subjects [29]. Consequently, music has been associated with significant improvements of life quality in dementia clinical cases [30]. Further, verbal communication in demented subjects may also be beneficially affected by listening and producing music [31]. Here, it should be noted that while improvements in behavioural and emotional health has been observed in several studies, beneficial effects of music therapy on cognitive attributes of dementia subject is contested [32]. Conversely, other studies have reported significant successes of music-based interventions in retarding cognitive decline in dementia subjects [33]. Various factors may be responsible for such discrepancies, as discussed in section 8.

## 5 Neurodevelopmental conditions: autism spectrum disorders (ASD) and attention deficit hyperactivity disorder (ADHD)

ASD is a common neurodevelopmental condition that primarily affects children under the age of six, leading to difficulties in social communication and behavioural dysfunctions. ASD involves altered intrinsic brain connectivity, particularly in the fronto-temporal and cortico-subcortical regions linked to social and verbal communication skills. The positive effects of music therapy in autistic subjects have been known for a while now [34]. Children with ASD have shown enhanced pitch perception, indicating brain plasticity and reorganization influenced by genetic factors. Listening to music is regarded as a rewarding activity for individuals with ASD, activating the dopaminergic response system and contributing to emotional regulation and mood [35].

Exposure to the appropriate pitch, tone and tempo of music enhances melodic memory and brain processing in children, while engaging in music activities stimulates multiple brain regions associated with memory, emotion, pleasure, and auditory processing [36]. Music therapy's effectiveness in autism subjects stems from top-down reward-based cortical modulation related to learning non-musical behaviours like social interaction, as well as bottom-up sensorimotor integration through sound and auditory-motor processing to enhance social communication skills [37]. Neuroimaging studies indicate the potential for brain plasticity and positive impacts on communication skills in autistic subjects. By harnessing the therapeutic power of music, individuals with ASD can experience improved social interactions, attention, and sensory processing, promoting their overall well-being and development. On-going research in this area continues to enrich our understanding of music-based interventions for individuals with autism. Interestingly, research on brain regions involved in speech and music development has shown that the arcuate fascicles, crucial for these processes, appear thicker on the right hemisphere than the left in children with autism. The right hemisphere is primarily responsible for interpreting musical sounds, while the left hemisphere is associated with speech. This finding offers a potential explanation for how music increases engagement among children with autism [38].

According to the diagnostic statistical manual of mental disorders (DSM), attention deficit hyperactivity disorder (ADHD) is a neurodevelopmental disorder commonly seen in children and adolescents, characterized by hyperactivity, hyperactivity, and an inability to focus [39]. ADHD is widely prevalent, and may elicit more frequently in male subjects. Thus, Venkata et al. found ADHD prevalence to be 11.33 % in an Indian community-based sample, with significantly more prevalence in males [40]. Stimulant medications are considered a primary treatment for ADHD, and their effectiveness in reducing the risk of retention among adolescents may be enhanced when combined with behavioural interventions, [41]. The relationship between music and ADHD brain is probably explained by the load theory of selective attention. According to this theory, focusing on a task involves balancing two cognitive mechanisms; early-selection, a deliberate choice of a target stimulus with a specific goal, and late-selection, involuntary attention drawn to a dominant stimulus. Cumulatively, music-based interventions result in appreciable benefits effects on self-regulation of ADHD subjects [42].

## 6 Mental health and mood disorders

It has been known since a long time that different types of music can activate differential emotional patterns in “normal” subjects with uncompromised mental health statuses [43]. With regards to clinical cases of mental health issues, music has been elicited to beneficially affect human behaviour and has consequently been examined for therapeutic applications against mental health conditions such as depression, stress and anxiety [44]. Meta-analyses of primary research has indicated the beneficial effects of music-based interventions, particularly in complementation of regularly practised clinical therapies, in alleviation of depression- and anxiety-like behaviour in human subjects of all ages [45]. The probable mechanisms underlying these effects seem to be modulation of the activities of brain regions associated with stress responses, as well as neoplastic changes in striatal regions, prefrontal cortex, amygdala, hippocampus and hypothalamus. Evidences from rodent models of early life stress concur with the hypothesis that music induces positive neuronal alterations, particularly with regards to hippocampal plasticity, for its effects of mitigation of behavioural and cognitive deficits [46]. Two of the most important substrates of music therapy in altering neuronal plasticity and alleviating mood disorders are dopaminergic signalling and brain-derived neurotrophic factor (BDNF). Indeed, music exposure is associated with anxiolytic consequences and fear abolishment via stimulation of BDNF signalling in anterior cingulate cortex [47]. Interestingly, Indian classical music has also been evidenced to reduce psychological stress and promote mindfulness and mitigate sleep problems, as reported by Sharma et al., who used EEG imaging in their studies on medical students [48]. Dingle and co-workers have summarized the various forms of music for their beneficial effects on emotions, arousal, social interactions, relaxation and cognition [49].

Considered by some as a neurodevelopmental disorder, schizophrenia is basically a behavioural disorder with varied aetiologies. Schizophrenia is characterized by disordered thoughts, feelings, and perceptions, and can present with acute symptoms like auditory or visual hallucinations, as well as chronic symptoms, including social withdrawal and memory issues. This condition may have significant detrimental effects on behavioural functions, such as creativity and ability to form relationships. Music therapy is widely regarded as a beneficial approach for managing various schizophrenia-related symptoms [50]. Indeed, recent evidences suggest music therapy as a very prominent component of multifactorial combinatorial therapeutic strategies against schizophrenia [51]. Such music-based interventions have been proposed to address motivational, emotional and social aspects of schizophrenics in a long-term manner. On the contrary, other studies indicate that positive impact of music therapeutic regimens may be reversible, and discontinuation may result in re-emergence and deterioration of the behavioural symptoms [52]. Moreover, duration and frequency of music-based therapeutic episodes heavily drive their effectiveness [53].

In addition to improvements of emotional and mental health statuses of clinical subjects of schizophrenia, music therapy has been found to significantly elevate their quality of life and social functioning [54]. Supplementing music-based interventions with regularly used clinical interventions has been shown to attenuate negative social and behavioural symptoms, allowing schizophrenic subjects to appreciably improve their interpersonal skills [55]. A recent meta-analysis revealed that music therapy has tremendous positive effects with regards to mitigation of social interaction deficits, anxiety-like behaviour, auditory hallucinations, and speech patterns [56]. These results are supported by magnetic resonance imaging data which indicate increased static functional connectivity between the limbic regions of the brain in schizophrenia patients [57,58]. Further research is however needed to gain a deeper understanding and their implications for music therapy in schizophrenia treatment. In particular, comprehensive long-term measurement of music therapy's effects must be studied in order to address all ambiguities.

## 7 Circadian rhythm disruptions

Circadian rhythm disruptions and sleep impairments are bi-directionally related to multiple molecular and cellular aspects of brain functional deficits in cognition, emotion, and behaviour [59,60]. An important aspect of music therapy interventions for chronic disorders, including neurological conditions, lies in its ability to beneficially affect circadian rhythm and sleep-wake cycle [61]. This is supported by a recent animal study which used zebrafish as a model system to assess the therapeutic effects of long-term music therapy on circadian disruption-induced cognitive and psychological impairments [62].

As reviewed recently by Sharma et al. [63], music is increasingly being recognized as an effective means of non-pharmacological intervention to ameliorate circadian disruptions, and thereby act as a neuroprotective measure targeting brain structural plasticity and other pathogenic mechanisms related to neurodegenerative states. Interestingly, the applications of music therapy for treatment of neurodegenerative disorders can be optimized by clock timing. This is in concurrence with a trial conducted by Theorell and co-workers [64], who illustrated that home-based music therapeutic intervention in dementia patients and their care-givers significantly reduced their salivary cortisol levels [64]. Further, slow “sedative” music has been noted to have beneficial effects on attributes of perceived sleep quality, duration, and latency in aged human subjects with circadian dysfunctions [65]. In concurrence, a meta-analytical study has revealed music as one of significantly effective interventional regimen for attenuating sleep dysfunctions (as assessed by the Pittsburgh Sleep Quality Index; PSQI) in both healthy subjects and those with varied medical conditions [66]. Further, music provision, in addition to other chronotherapeutic strategies, has been proposed as a means to rescue dysfunctional sleep-wake characteristics in critically ill patients suffering from delirium [67]. Lastly, in an interesting study, Ceccato and Roveran [68] proposed that group-based music therapy before evening meals may reduce pre-meal anxiety in anorexia nervosa patients, indicating its possible utility in attenuation of circadian disruptions.

## 8 Future prospects

Music therapy has been a profoundly sought after intervention for neurological disorders. Through the development of synchronous rhythm, motor imitation, and joint attention, music therapy is thought to support preverbal communication [69]. In patients of schizophrenia, non-verbal social communication that can be introduced by the use of music therapy can be particularly useful [54]. In the case of ASD, patients showed intact emotion recognition from music, as expressed in their behavioral ratings, and in typical brain processing of emotional music, with activation of limbic and paralimbic areas, including reward regions [70]. Patients with dementia can remember music despite having significant memory impairments, and music can help people retain episodic memories, even if the music is unrelated to the events being remembered. Additionally, music has been employed therapeutically to encourage social bonding in these patients [71]. Improvements in frontal lobe functions (cognitive flexibility, processing speed, attention, and working memory) is a relevant outcome of music-based intervention for enhancement of attention and executive functions [20]. Further, studies in ADHD patients have revealed significant decreases in cortisol levels, indicating lowered depression and stress levels. This was seen to occur in tandem with the responses from other neurophysiological factors like blood pressure, heart rate and hormones [72]. Importantly, no specific adverse effects were observed in any of the studies, and almost all patients tolerated music therapy well.

In spite of potential multimodal therapeutic aspects against different neurological disorders, there is insufficient evidence for the exact nature of music-based therapy [73]. Many studies on music therapy employed small sample sizes, which may limit the generalizability of the findings. Another drawback could be the lack of control groups which makes it difficult to establish a cause-and-effect relationship between therapy and the observed outcomes. Further, heterogeneity of the study populations is one of the top reasons for the diverse outcomes in all interventional studies. All patients with the disorders have varying degrees of impairment and symptoms. Hence, interventions may vary widely, depending on the patient's needs, the therapist's training, and the context in which the therapy is being delivered. Further, heterogeneity at the genetic level, coupled with low samples sizes, may lead to erroneous conclusions (e.g., whether experience-dependent or not) regarding the neuroplasticity-related effects induced by music exposure. Along similar lines, gender-based differences in the outcomes of music therapy for neurological conditions need to be addressed in detail. Likewise, not many studies have comprehensively analysed the impact of music on the young nervous system [74].

Other significant limitations of studies which have focussed on music therapy for neurological disorders include absence of standardized interventional protocols, disregard for cultural perspectives, and inadequacies in addressing the influences from confounders such as general environmental conditions in the hospitals/care centres, background noise, patient noise sensitivity, therapists' attitudes, etc. Further, a thorough comparison between the effects of auditory (listening), vocal (singing), motor (listening/singing coupled with dancing) and sensorimotor (instrument playing) attributes of music intervention, as well as their complementation actions, need to be assessed in a disease- and patient-specific manner. Likewise, studies should also seek to understand how patients' cultural and musical orientations shape the treatment processes. By identifying these cultural specificity issues, one could learn how to individualize the provision of music therapy in terms of patients’ cultural backgrounds and life experiences. Other key aspects of music therapy for neurological disorders which have not been optimally evaluated in a majority of studies are the underlying mechanisms and the longevity of the beneficial effects (particularly in acute clinical settings). Core neurophysiological mechanism behind the therapy and effect of music should be explored in as much as detail for a better understanding of the pathways involved; although this may be challenging under clinical and pre-clinical settings. Lastly, in order to better understand the effectiveness of the treatment course of music therapeutic regimens, future trials need to investigate their complementation with other approaches, including pharmacological and behavioural ones. Interaction of music and other therapies may have added effects on neuroplasticity enhancement and functional abilities of patients.

## 9 Conclusions

In conclusion, music application in neurological disorders is increasingly being regarded as a promising and diverse field for therapeutic approaches. This form of therapy has been found to be effective in varied conditions, including those affecting the developing brain such as autism and ADHD, as well as ageing-related impairments such as Parkinson's disease and dementia. Consequently, music's impact on influencing neuroplasticity in multiple brain regions/systems, developing and optimizing the mental, emotional, behavioural, sleep and cognitive functions, as well as increasing quality of life reveals its opportunity to be harnessed as a potent non-invasive and non-pharmacological treatment alternative. Nonetheless, further high-quality studies are required because the body of research on music therapy is presently insufficient. Refinement of music applying methods and better understanding of the underlying mechanisms and pathways should be the goals for future research. In this regard, we hope that the present review forms a suitable research platform outlining the pertinence of music interventions, and the need forsynchronizing and extending the experimentation on a larger scale in order to gain adequate understanding of its therapeutic potential for neurological disorders.

## Ethical approval and informed consent statements

Not applicable, as no primary data were generated.

## Data availability statement

The authors report there are no competing interests to declare.

## Funding

Deputyship for Research & Innovation, Ministry of Education, Saudi Arabia - Project Number: ISP23-101.

## CRediT authorship contribution statement

**Medha Ramaswamy:** Writing – review & editing, Writing – original draft, Visualization, Validation, Software, Resources, Methodology, Investigation, Formal analysis, Data curation, Conceptualization. **Johann Laji Philip:** Writing – review & editing, Writing – original draft, Visualization, Validation, Software, Resources, Methodology, Investigation, Formal analysis, Data curation, Conceptualization. **Vijayan Priya:** Writing – review & editing, Visualization, Validation, Methodology, Investigation, Formal analysis, Data curation. **Snigdha Priyadarshini:** Writing – review & editing, Writing – original draft, Visualization, Validation, Software, Resources, Methodology, Investigation, Formal analysis, Data curation, Conceptualization. **Meenakshi Ramasamy:** Writing – review & editing, Writing – original draft, Visualization, Validation, Software, Resources, Methodology, Investigation, Formal analysis, Data curation, Conceptualization. **G.C. Jeevitha:** Writing – review & editing, Visualization, Validation, Methodology, Investigation, Formal analysis, Conceptualization. **Darin Mansor Mathkor:** Writing – review & editing, Visualization, Validation, Methodology, Investigation, Formal analysis, Conceptualization. **Shafiul Haque:** Writing – review & editing, Writing – original draft, Visualization, Validation, Supervision, Software, Resources, Project administration, Methodology, Investigation, Funding acquisition, Formal analysis, Data curation, Conceptualization. **Fatemeh Dabaghzadeh:** Writing – review & editing, Visualization, Validation, Methodology, Investigation, Formal analysis, Conceptualization. **Pratik Bhattacharya:** Writing – review & editing, Writing – original draft, Visualization, Validation, Supervision, Software, Resources, Project administration, Methodology, Investigation, Formal analysis, Data curation, Conceptualization. **Faraz Ahmad:** Writing – review & editing, Writing – original draft, Visualization, Validation, Supervision, Software, Resources, Project administration, Methodology, Investigation, Formal analysis, Data curation, Conceptualization.

## Declaration of competing interest

The authors declare that they have no known competing financial interests or personal relationships that could have appeared to influence the work reported in this paper.
